# Advances of Fibroblast Growth Factor/Receptor Signaling Pathway in Hepatocellular Carcinoma and its Pharmacotherapeutic Targets

**DOI:** 10.3389/fphar.2021.650388

**Published:** 2021-04-15

**Authors:** Haijun Wang, Jie Yang, Ke Zhang, Jia Liu, Yushan Li, Wei Su, Na Song

**Affiliations:** ^1^ Key Laboratory of Clinical Molecular Pathology, Department of Pathology, The First Affiliated Hospital of Xinxiang Medical University, Xinxiang, China; ^2^ School of Basic Medical Sciences, Xinxiang Medical University, Xinxiang, China; ^3^ School of Public Health, Xinxiang Medical University, Xinxiang, China; ^4^ Institute of Precision Medicine, Xinxiang Medical University, Xinxiang, China

**Keywords:** fibroblast growth factor, fibroblast growth factor receptor, signaling pathway, hepatocellular carcinoma, pharmacotherapeutic targets

## Abstract

Hepatocellular carcinoma (HCC) is a type of primary liver cancer with poor prognosis, and its incidence and mortality rate are increasing worldwide. It is refractory to conventional chemotherapy and radiotherapy owing to its high tumor heterogeneity. Accumulated genetic alterations and aberrant cell signaling pathway have been characterized in HCC. The fibroblast growth factor (FGF) family and their receptors (FGFRs) are involved in diverse biological activities, including embryonic development, proliferation, differentiation, survival, angiogenesis, and migration, etc. Data mining results of The *Cancer* Genome Atlas demonstrate high levels of FGF and/or FGFR expression in HCC tumors compared with normal tissues. Moreover, substantial evidence indicates that the FGF/FGFR signaling axis plays an important role in various mechanisms that contribute to HCC development. At present, several inhibitors targeting FGF/FGFR, such as multikinase inhibitors, specific FGFR4 inhibitors, and FGF ligand traps, exhibit antitumor activity in preclinical or early development phases in HCC. In this review, we summarize the research progress regarding the molecular implications of FGF/FGFR-mediated signaling and the development of FGFR-targeted therapeutics in hepatocarcinogenesis.

## Introduction

Hepatocellular carcinoma (HCC) accounts for 85–90% of primary liver cancer and is commonly associated with underlying chronic liver disease arising from viral infection, metabolic disorders, or alcohol abuse (Best et al., 2017). Primary liver cancer has become the sixth most common cancer in terms of incidence and is the third cause of cancer-related mortality worldwide (Sung et al., 2021). Despite some improvements in overall survival, the prognosis of patients with HCC remains poor; the ratio of estimated number of deaths to incident cases is 75% (Craig et al., 2019). According to the Global Cancer Observatory data, the mortality of HCC will be 1.28 million in 2040 vs. 0.78 million in 2018 (a 64.3% increase), and the incidence will increase by 61.9% (from 1.36 to 0.84 million). The incidence of HCC has a certain gender orientation, with males having a higher risk. The ratio of HCC incidence is 13.9/4.9 per 100,000 people in the world, and the value is 27.6/9.0 in China. Eastern Asia is recognized as the traditionally highest-risk region, especially Japan and China.

Generally, surgery is the predominant therapy for HCC (Bruix et al., 2005; Llovet et al., 2015). However, the outcome is poor, and the risk of recurrence and metastasis remains high even after surgery. Biological therapy is a promising therapy in a series of cancers that targets the biomarkers or signaling pathway. The development of HCC is a multistep process, in which epigenetic changes and genetic alterations accumulate in HCC cells, including mutations and DNA amplification variations, which result in cell signaling pathway variation, ultimately leading to the high heterogeneity of HCC (Moeini et al., 2012).

The fibroblast growth factor (FGF) family and their receptors (FGFRs) play crucial roles in regulating physiologic cellular processes, and they contribute to embryonic development, proliferation, differentiation, survival, angiogenesis, and migration (Turner and Grose, 2010; Yun et al., 2010; Sandhu et al., 2014). The deregulation of FGF signaling is frequently observed in HCC and liver cirrhosis, as well as viral hepatitis. Evidence shows that the FGF family and FGFRs can be used to elucidate the development and progression of HCC, even its treatment (Motoo et al., 1993; Beenken and Mohammadi, 2009). In this review, we summarize the current research progress regarding FGF/FGFR signaling in hepatocarcinogenesis and the potential pharmacological applications of FGFRs in HCC.

### Overview of FGFS and FGFRS

FGFs are polypeptide growth factors that regulate diverse biological activities, ranging from cell growth, development, differentiation, wound repair to angiogenesis and tumorigenesis (Beenken and Mohammadi, 2009; Ornitz and Itoh, 2015). The first FGF-like factor with mitogenic activity was discovered in 1939 and isolated in the 1970s (Gospodarowicz et al., 1974). The human-mouse FGF family comprises 22 related proteins with similar structure and evolution, FGF1–FGF23 (Beenken and Mohammadi, 2009; Cheng et al., 2011). However, FGF19 only exists in humans, not in mice; it is highly homologous with FGF15 in mice, which are also referred as FGF15/FGF19 (Itoh, 2010). The FGF family is divided into seven subfamilies according to gene evolution analysis: FGF/1/2/5, FGF3/4/6, FGF7/10/22, FGF8/17/18, FGF9/16/20, FGF11/12/13/14, and FGF15/19/21/23. FGF members are also classified into paracrine, endocrine, and intracrine FGFs on the basis of their mechanisms of action. The FGF11 subfamily belongs to intracrine FGFs, the FGF19 subfamily is recognized as endocrine FGFs, while the other 15 members of FGFs are paracrine cytokines (Itoh and Ornitz, 2008; Itoh and Ornitz, 2011).

**FIGURE 1:**
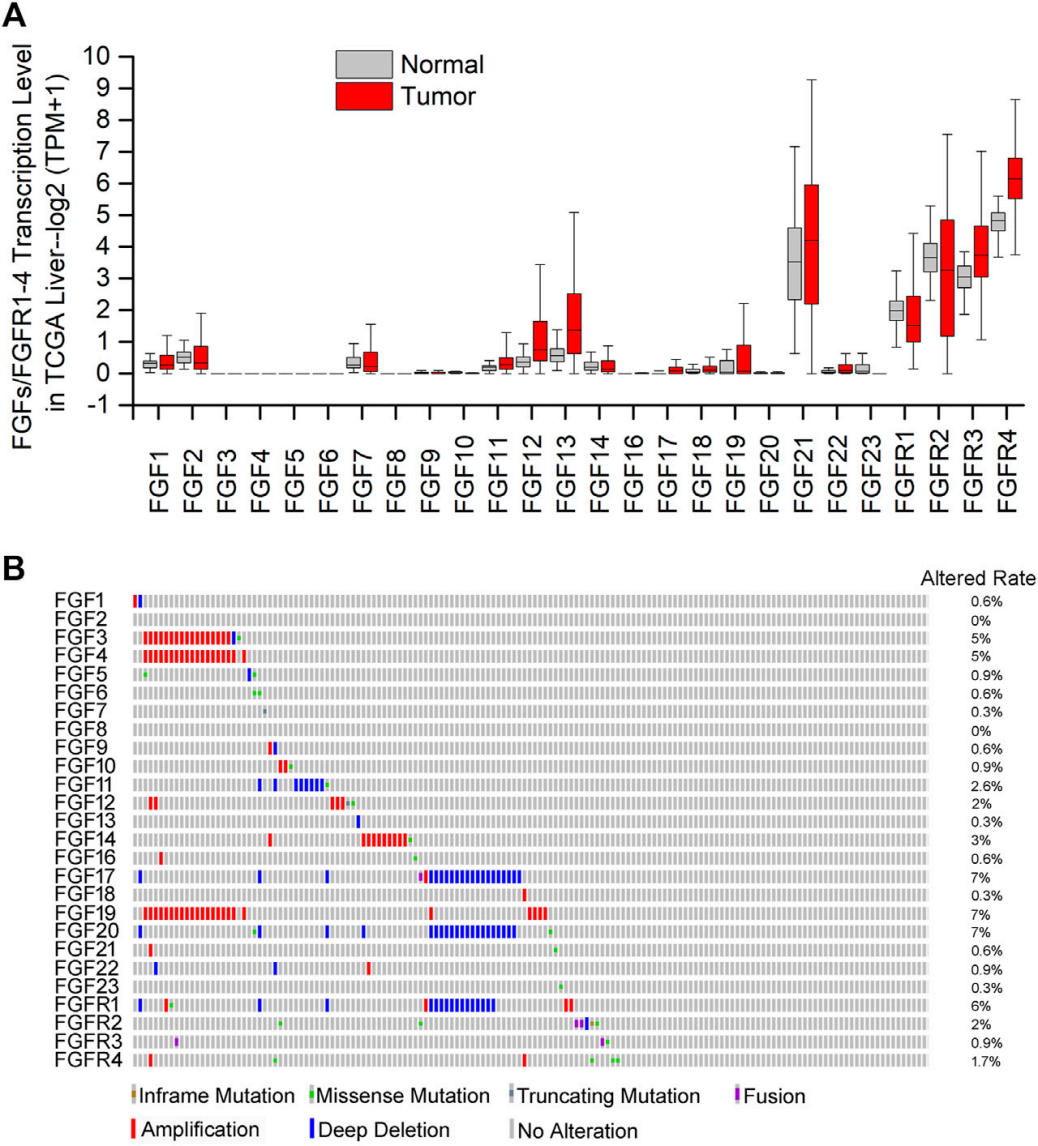
Gene transcription level and mutation status of FGFs/FGFRs in HCC patients were investigated with TCGA database analysis **(A)** The transcription levels of FGF1–23 and FGFR1–4 was investigated with GEPIA platform (http://gepia.cancer-pku.cn/) between HCC tumor vs. normal samples based on TCGA database **(B)** The mutation status of FGF1–23 and FGFR1–4 was displayed based on cBioPortal database (https://www.cbioportal.org/). Altered rate: altered/total profiled.

FGFs, especially paracrine and endocrine FGFs, transduce cell signals via binding to and activating tyrosine kinase receptors (FGFRs) on the surface of the target cells (Eswarakumar et al., 2005). The human FGFR gene family consists of four members, FGFR1–4. Except for FGFR4, the other types of FGFRs encode two alternative splicing variants (b and c). Therefore, seven forms of FGFR proteins with distinct ligand-binding specificity exist in humans, FGFRs 1b, 1c, 2b, 2c, 3b, 3c, and 4 (Ornitz and Itoh, 2015). In the binding of FGFs–FGFRs, heparin/heparan sulfate (HS) and klotho family members (α, β) are needed as co-receptors (Ornitz and Itoh, 2015). Generally, paracrine FGFs bind to and activate FGFRs with heparin/HS, and mediate multiple developmental and physiological processes. By contrast, endocrine FGFs (FGF15/19/21/23), with a low affinity to HS, usually require the klotho proteins for high affinity binding and activating FGFRs in multiple metabolic processes and carcinogenesis (Itoh, 2010). Research reports that only a few endocrine FGF affect the progression of HCC (Ornitz and Itoh, 2015). The specific interaction of FGFs–FGFRs with cofactors activates several intracellular cascades, including Ras/MAPK, PI3K/Akt, and PLCγ/PKC pathways, to regulate target genes transcription (Katoh and Nakagama, 2014). Abnormalities of FGFs/FGFRs will lead to many diseases and are considered as a risk factor in the development of cancer (Itoh, 2010; Touat et al., 2015; Mikhaylenko et al., 2018). The gene transcription level (Figure 1A) and mutation status of (Figure 1B) FGFs/FGFRs were also investigated in HCC patients based on GEPIA2 and cBioPortal database (Cerami et al., 2012; Gao et al., 2013; Tang et al., 2017; Tang et al., 2019). As shown in Figure 1A, the transcription levels of FGF1, 2, 7, 11–13, 17–19, and 21–22 and FGFR1–4 in hepatocellular tumor tissues are higher than those in normal tissues (Figure 1A); In addition, gene mutations, fusions, and copy number amplification of FGFs/FGFRs are closely related to the occurrence of HCC. As shown in Figure 1B, FGF1, 3–7, 9–22 and FGFR1-4 all have different degrees of genetic changes, especially gene copy number amplification and deep deletion. Moreover, the FGF1 subfamily and FGF18 could promote angiogenesis, FGF15/19 binding with FGFR4 can be used as a potential biomarker in HCC. Above all, there is a growing interest in targeted agents based on FGF and FGFR for HCC, relevant clinical trials are being carried out, such as brivanib, dovitinib, FGF401, and BLU-554 (Katoh and Nakagama, 2014; Katoh, 2016; Spallanzani et al., 2018; Lu et al., 2019; Subbiah and Pal, 2019; Weiss et al., 2019).

### FGF1 Subfamily

FGF1 (aFGF) and FGF2 (bFGF) are the first discovered FGF family members, which were originally isolated from the brain and pituitary gland (Gospodarowicz, 1975; Gambarini and Armelin, 1982; Lemmon and Bradshaw, 1983). The amino acid homology between FGF1 and FGF2, which belong to the FGF1 subfamily, is as high as 55%. Due to the lack of a signal sequence and no secretion, FGF1 and FGF2 cross the membrane through a process facilitated by binding the cell surface and extracellular matrix (ECM) heparan sulfate (HS). FGF1 subfamily members function through an autocrine manner, inducing HCC proliferation, invasion, and angiogenesis (Kin et al., 1997). The expression of FGF1 and FGF2 is induced in chronic liver diseases, and their expression levels are increased in more advanced tumor stages (Jin-no et al., 1997; Poon et al., 2001; Asada et al., 2003; Cheng et al., 2011; Sandhu et al., 2014).

Compared with FGF1, the oncogenetic effect of FGF2 in various tumors has been studied (Baird and Klagsbrun, 1991; Beenken and Mohammadi, 2009; Cheng et al., 2011; Sandhu et al., 2014; Itoh et al., 2016). In mice transplanted with HCC cells, anti-basic FGF antibody injection slowed down and suppressed tumor growth. The deregulation of FGFR3 with specific siRNA inhibits the HCC cell growth, indicating that FGF2 and its receptors (FGFR3) are essential for HCC proliferation (Poon et al., 2001; Midorikawa et al., 2003). Several studies suggested that FGF2 expression is correlated with the invasiveness and postsurgical survival of HCC (Poon et al., 2001; Poon et al., 2003).

HCC is a devastating disease with high angiogenesis. FGF1 and FGF2 are correlated with increased sinusoidal capillarization, which is involved in tumor angiogenesis (Motoo et al., 1993). It was supported that blocking FGF2 with vascularization inhibitors leads to a significant reduction in tumor size (Wang and Becker, 1997). FGF2 and VEGF acted synergistically in tumor angiogenesis to accelerate tumor progression, at least on the angiogenic maintenance of tumors in HCC patients (Wang and Becker, 1997; Yoshiji et al., 2002). Furthermore, increased levels of FGF2 were detected in the serum of cancer patients who have become resistant to VEGF-targeted therapy, which suggests the indirect role of FGF2 in angiogenic resistance. Thus, dual targeting of VEGF/FGF is a considerable strategy to circumvent therapy resistance (Casanovas et al., 2005). The latest research claimed that FGF2 single nucleotide polymorphisms (SNPs) rs308379 A allele could be regarded as an independent poor prognostic factor for overall survival in patients with HBV-associated HCC by multivariate Cox analysis (Kim et al., 2019b). Taken together, FGF1 and FGF2 are believed to be of great importance in the development of HCC.

### FGF8 Subfamily

FGF8, FGF17, and FGF18 are members of the FGF8 subfamily with strong homology and evolutionary relationship (Gauglhofer et al., 2011). Four FGF8 isoforms exist for alternative splicing. These FGF8 variants, FGF17, and FGF18, which act as local paracrine molecules, are presumed to bind and activate FGFR2, FGFR3, and FGFR4 (Zhang et al., 2006; Beenken and Mohammadi, 2009). The FGF8 subfamily exerts oncogenic effects in HCC. According to statistics, at least one member of the FGF8 subfamily and/or their receptors is upregulated to facilitate cell survival and angiogenesis via activating ERK in the majority of HCC cases studied (Gauglhofer et al., 2011). FGF8, FGF17, and FGF18 seem to be important drivers of proliferation, malignant behavior, and neovascularization in advanced stages of HCC. The proliferation and neovascularization of myofibroblasts (MFs) cultured from HCCs can be induced by additional FGF8 subfamily members via the modulation of VEGF pathways (Alessandri et al., 1998). The administration of FGF8, FGF17, or FGF18 could resist apoptosis and enhance the survival of serum-starved tumor cells, including HCC-1.2, HepG2, and Hep3B cells, the mechanism studies have found that these effects were mediated by ERK and AKT/mTOR signaling pathways (Gauglhofer et al., 2011; Liu et al., 2015a). Therefore, the role of FGF8 subfamily members in the occurrence and development of HCC should not be neglected. Most studies on the current FGF8 subfamily in HCC mainly focused on FGF8 and FGF18. We will elaborate the activity and mechanism of these FGFs in HCC, as outlined in the following.

FGF8 was first identified in the SC-3 cell line, which is a mammary carcinoma cell line of Shionogi mouse and induced by androgen (Chen et al., 2016; Linscott and Chung, 2016). FGF8 was found to be overexpressed in several solid cancers, including HCC, but rarely detected in normal adult tissues (Liu et al., 2015a). Zou et al. confirmed that FGF8 is one of the advanced markers in stage III–IV HCC tumors with The Cancer Genome Atlas (TCGA) data and *in vitro* as well as *in vivo*. Their study also demonstrated that increased FGF17 plays a prediction role in stage II–IV HCC tumors. FGF19 and FGF4 are significantly upregulated in stage I and function as early markers (which will be described in detail in the FGF19 subfamily section) (Zou et al., 2018). The overexpressed or exogenous recombinant FGF8 promotes HCC cell growth by mediating the YAP1/EGFR axis. Meanwhile, exogenous recombinant FGF8 plays a critical role in the resistance to EGFR inhibitor gefitinib in HCC cells, but not to other anticancer chemotherapeutic drugs, such as doxorubicin, 5-Fu, paclitaxel, RAD001, and oxaliplatin (Liu et al., 2015a).

FGF18 is conserved among different species, including humans, mouse, and rats, whose amino acid identity is 99% (Hu et al., 1998; Ohbayashi et al., 1998; Haque et al., 2007). Similar to FGF2, FGF18 also acts as a mitogen in embryonic limb development and is especially required in the development of bone, cartilage, and alveologenesis (Hajihosseini and Heath, 2002; Liu et al., 2007; Hung et al., 2016; Zhang et al., 2019a; Wang et al., 2019; Antunes et al., 2020; Hagan et al., 2020). FGF18 plays a key role in regulating the biological activity of tumor cells and immediately surrounding tissue cells of the tumor microenvironment via multiple signaling pathways (Shimokawa et al., 2003; Sonvilla et al., 2008; Gauglhofer et al., 2011; Zhang et al., 2019a; Zhang et al., 2019b; Jomrich et al., 2019). Pronounced overexpression of FGF18 accelerates tumorigenesis via mediating cell proliferation, invasion, and angiogenesis, which is correlated with poor overall survival in patients and has been addressed in multiple types of cancers, such as HCC, colorectal carcinomas, ovarian cancers, and gastric cancer (Gauglhofer et al., 2011; Koneczny et al., 2015; El-Gendi et al., 2016; Flannery et al., 2016; Zhang et al., 2019a; Zhang et al., 2019b; Jomrich et al., 2019; Kulbe et al., 2019). Animal studies also confirmed the oncogenic role of FGF18. FGF18 overexpression in the liver of transgenic mice induced a marked increase in liver weight and hepatocyte proliferation (Reinhold and Naski, 2007). Significantly increased expression levels of FGF18 were detected in rat hepatocellular adenoma and carcinoma via the autocrine pathway. Studies demonstrated that the oncogenic role of FGF18 could be directly suppressed by miR-139 in HepG2 and Huh7 cells, the downregulation of FGF18 is related to the inhibition of cell invasion, migration, angiogenesis and promotion of apoptosis (Yang et al., 2017). Similarly, silencing FGF18 with specific siRNA decreased the viability and clonal proliferation of HCC cell lines, but elevated apoptotic activity in HCC cell lines (Gauglhofer et al., 2011).

Of note, various growth factor systems in the liver tumor microenvironment of inflammatory cells, small vessels, MFs, and ECM components will accelerate hepatocarcinogenesis (Sagmeister et al., 2008). FGF8 subfamily members are related to tumor–stroma communication. FGF18 and FGF17 contribute to replicative DNA synthesis in MFs. Moreover, all FGF8 subfamily members participate in the tube formation of endothelial cells, which is essential for neoangiogenesis (Gauglhofer et al., 2011). FGF18 increases protein synthesis and cell growth to induce HCC vascularization in liver-specific endothelial cells, which are associated with the function of ribosomal protein RPS6 (Clevers, 2000). Consistently, FGF18 expression and secretion are upregulated in a high-RPS15 A-expression HCC tumor microenvironment; FGF18 interacts with FGFR3 and contributes to angiogenesis by inducing the Wnt/β-catenin signaling pathway in endothelial cells (Guo et al., 2018). Similar to other FGFs, FGF18 also prefers to induce the formation of new blood vessels in HCC via directly or indirectly regulating VEGF of tumor cells and surrounding tissue cells of the tumor microenvironment. Taken together, FGF18 contributes to the progression of HCC via paracrine and autocrine ways.

### FGF19 Subfamily

The FGF19 subfamily consists of FGF15/19, FGF21, and FGF23. Although Fgf15/19, FGF21, and FGF23 have only about 22–35% amino acid identity, phylogenetic and gene locus analyses suggested that they belong to one subfamily (Itoh and Ornitz, 2004, 2008; Mikhaylenko et al., 2018). FGF15 and FGF19 are the mouse and human orthologs, respectively, which share 53% amino acid homology. We refer to them collectively as FGF15/19 unless referring to a specific ortholog (Itoh and Ornitz, 2011). FGF19 subfamily members have been identified in vertebrates but not invertebrates (Itoh and Ornitz, 2004; Itoh, 2007). In contrast to other FGFs, FGF19 subfamily members act in an endocrine way because of the low binding affinity to HS, which facilitates their diffusion over long distances from the tissue of production and the secretory area, and act as endocrine hormones (Zhang et al., 2006; Goetz et al., 2007; Itoh, 2010; Tulin and Stathopoulos, 2010; Beenken and Mohammadi, 2012). Endocrine FGFs need the assistance of klotho proteins to adjust the interaction of FGFs and corresponding FGFRs to mediate biological activity via triggering classical FGF pathways (Choi et al., 2006; Beenken and Mohammadi, 2012; Kuro-o, 2012). FGF23 activates FGFR1c via binding with *a*-klotho, which originates from the bone, secreted by osteocytes/osteoblasts, but is responsible for phosphate homeostasis and vitamin D administration in the kidney (Urakawa et al., 2006; Fon Tacer et al., 2010; Ersoy, 2014; Fukumoto, 2019; Pereira et al., 2019). Targeted ablation of FGF23 in mice causes severe hyperphosphatemia, along with osteoporosis, vascular calcification, atherosclerosis, sterility, and survival reduction but no significant effect on liver diseases (Shimada et al., 2004).

Compared with FGF23, FGF15/19 and FGF21 need *ß*-klotho (KLB) as co-receptor to bind to their FGFRs. Both of them can activate the IIIc isoform of FGFR1, two and 3. However, only FGF19 activates FGFR4 *in vitro*. The abundant expression of *ß*-klotho in the liver indicates that FGF15/19 and FGF21 act in the liver, which has been confirmed by multiple studies (Kharitonenkov et al., 2008; Yang et al., 2012a; Ding et al., 2012; Schumann et al., 2016; Agrawal et al., 2018; Ritchie et al., 2020). FGF19 is involved in postprandial gut–liver communications and acts as a growth factor for hepatocytes as well as hepatic bile acid synthesis (Kir et al., 2011). However, numerous pieces of evidence indicated that FGF15/19 is not physiologically expressed in the liver, but pathological FGF19 expression was detected in liver tissues of patients with liver diseases, including hepatitis C virus cirrhosis, cholestasis, and HCC (Inagaki et al., 2007; Naugler et al., 2015). FGF15/19 function as a driver for HCC (Miura et al., 2012; Mellor, 2014; Repana and Ross, 2015; Li et al., 2016b; Cui et al., 2018; Lin et al., 2019b Maeda et al., 2019; Raja et al., 2019). FGF21 is predominantly produced by the liver and also expressed in adipose tissue, skeletal muscle, pancreas, and many other organs. FGF21 is now considered as a key regulator of stress response in humans (Luo et al., 2017; Wu et al., 2017). Under stress conditions, elevated circulating FGF21 levels mostly appear to be derived from the liver, which has been confirmed in a series of liver-related disease models, such as liver inflammation; liver injury elicited by ethanol, drugs, or ischemia/reperfusion; liver regeneration; and hepatocarcinogenesis (Yang et al., 2013a; Ye et al., 2014; Ye et al., 2016; Desai et al., 2017; Wu et al., 2017; Ritchie et al., 2020). Taken together, the liver is recognized as a major direct or indirect target organ for FGF15/19 and FGF21 because of their expression in the liver with physiological and pathological conditions. Meanwhile, *ß*-klotho and FGFR4 are predominantly expressed in the liver. Therefore, FGF15/19 or FGF21, FGFR4, and *ß*-klotho coreceptor signaling system play as key regulators in hepatocarcinogenesis (Alvarez-Sola et al., 2017). Here, we will review the role of FGF15/19 and FGF21 on HCC in detail.

Abnormal FGF15/19-FGFR4 signaling pathway is an important cause of HCC, which is a complex and strongly heterogeneous type of cancer. Through genomic analysis, FGF19 gene amplification has been characterized in a subset of HCC tumors from patients with poor prognosis, and the expression of FGF19 is almost 48% in resected HCCs. After curative resection, circulating levels of FGF19 in HCC patients decreased. Furthermore, FGF19 protein expression in HCC tissues is significantly related to larger tumor size, advanced disease stage, and early recurrence (Sawey et al., 2011; Miura et al., 2012). In childhood hepatoblastoma, FGF19 gene amplification is not as prevalent as in adult HCC (approximately 5%) but significantly related to the degree of aggressiveness (Elzi et al., 2016). Animal studies showed that muscle-specific FGF19 transgenic mice at 10 months of age were used to generate liver cancer formation (Nicholes et al., 2002). And FGF15 participates in liver regeneration after partial hepatectomy inducing hepatocellular proliferation, FGF15^−/−^ mice showed lesser and smaller tumors, and histological neoplastic lesions were also smaller than those in FGF15^+/+^ animals (Uriarte et al., 2015). The above studies proved that high levels of FGF19 contribute to the development of HCC, function as an independent prognostic factor for survival, and may predict early recurrence of HCC after curative hepatectomy (Miura et al., 2012; Li et al., 2016b; Alvarez-Sola et al., 2017; Gao et al., 2017; Cui et al., 2018; Raja et al., 2019). The function of FGF19 is dependent on the expression of FGFR4 and KLB (Kurosu et al., 2007; Lin et al., 2007; Fon Tacer et al., 2010). Increased expression of FGFR4 or KLB and the decreased expression of SULT2A1 and KNG1 (FGF19 signaling repression targets) have also shown to be associated with shorter survival or development of multiple tumors, respectively (Desnoyers et al., 2008; Ho et al., 2009; French et al., 2012; Lin et al., 2019b; Raja et al., 2019). FGF19 may enhance its biological effects on HCC by activating multiple growth factor pathways. FGF19 positively regulates the expression of the EGFR ligand amphiregulin and connective tissue growth factor (CTGF) to regulate the growth and survival of HCC cells (Castillo et al., 2006; Castillo et al., 2009; Urtasun et al., 2011; Latasa et al., 2012; Uriarte et al., 2015). FGF19 increases the invasive capabilities of human HCC cell lines by promoting epithelial–mesenchymal transition (EMT) via a GSK3β/β-catenin pathway (Miura et al., 2012; Zhao et al., 2016). Oncogenomic screening also demonstrated that the amplification and overexpression of FGF19 occurs along with those of CCND1, known as an oncogene in HCC (Sawey et al., 2011). Non-cell-autonomous activation of IL-6/STAT3 signaling is involved in FGF19-driven hepatocarcinogenesis (Zhou et al., 2017).

As a risk factor to HCC development, the overexpression of FGF19 was also detected in patients with hepatitis C virus cirrhosis and biliary cirrhosis. Hepatic tissue protein of FGF19 and FGFR4 is significantly correlated with histopathologic changes from fatty liver to HCC via regulating the epithelial cell adhesion molecule (Li et al., 2016b). Ileum-derived FGF15/FGF19 could contribute to hepatocarcinogenesis in the presence of pro-tumorigenic conditions, such as chronic viral infection and alcohol consumption. FGF15 overexpression accelerates fibrosis and hepatocarcinogenesis via the upregulation of amphiregulin (AR), TGF-β, and CTGF (Uriarte et al., 2015). In a nonalcoholic steatohepatitis (NASH)-HCC mouse model, FGF15/FGFR4 signaling plays a critical role in HCC initiation and development via stimulating EMT and Wnt/β-catenin signaling (Cui et al., 2018). The findings lend support to the pro-tumorigenic potential of FGF15/FGF19 in the metabolic disorder microenvironment and address the importance of the role that FGF15/FGF19 play in HCC development.

**FIGURE 2:**
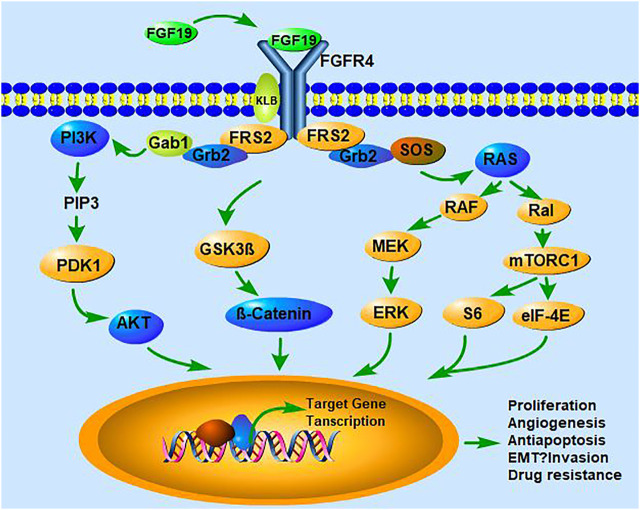
FGF19–FGFR4 signaling pathways in HCC. FGF19, FGFR4, and KLB comprise the complex, and the activated complex stimulates a cascade of pathways, including the PI3K/AKT, RAS/RAF/MAPK, RAS/Ral/mTORC1, and GSK3β/β-catenin pathway. FGF19–FGFR4–KLB (β-klotho) signals are involved in proliferation, angiogenesis, anti-apoptosis, EMT, invasion, and drug resistance in target cells.

Taken together, the FGF15/19-FGFR4 pathway contributes to the development of HCC. Its stimulation either through the amplification or overexpression of FGF15/19 in human HCC cells and tissues and its antitumoral effects by knocking down FGF19, FGFR4, or KLB or by the overexpression of dominant-negative FGFR4 variants in liver cancer cells *in vitro* and *in vivo* models have been shown to impact HCC cell proliferation, survival, EMT, migration, invasion, and drug resistance (French et al., 2012; Miura et al., 2012; Mellor, 2014; Repana and Ross, 2015; Alvarez-Sola et al., 2017; Gao et al., 2017; Cui et al., 2018; Kang et al., 2019; Kim et al., 2019a; Lin et al., 2019b; Raja et al., 2019; Weiss et al., 2019). The FGF19–FGFR4–KLB signal cascade was amplified via the activation of PI3K/AKT, RAS/RAF/MAPK, RAS/Ral/mTORC1, and GSK/β-catenin cell signaling pathways to further mediate the development and progression of HCC (Figure 2) (Wan et al., 2016). High levels of FGF19 can be used as an independent prognostic factor for survival and may predict early recurrence of HCC after curative hepatectomy (Lin et al., 2019b). Meanwhile, targeting FGF19 by shRNA or anti-FGF19 antibody neutralization or FGFR kinase inhibitors, LY2874455, BLU-554, and INCB06207, has been shown to inhibit the clonogenicity and tumorigenicity of FGF19 abnormal HCC (Hagel et al., 2015; Repana and Ross, 2015; Gao et al., 2017; Joshi et al., 2017; Kim et al., 2019a; Hatlen et al., 2019; Weiss et al., 2019). FGF19 could be a promising molecular target for the treatment of human HCC.

FGF21 consists of 210 amino acids in mice and 209 amino acids in humans (Motoo et al., 1993). Similar to FGF19/15 and FGF23, the effects of FGF21 are limited by the tissue-specific expression and signaling of different isoforms of FGFRs and KLB. As an inducible stress-sensing hepatokine, FGF21 expression is associated with the loss of normal functional capacity of hepatocytes due to pathogenic processes. In the normal condition, the expression of FGF21 is only detectable at a low level in the liver. However, the expression of hepatic FGF21 is increased significantly in liver diseases, such as partial hepatectomy and regeneration, hepatosteatosis, and irreversible hepatic damage from chronic hepatitis, cirrhosis, and even chemical (DEN treatment) and genetic-induced hepatocarcinogenesis (disruptions in LKB1, p53, MST1/2, SAV1, and PTEN) in mouse models and human patient samples (Yang et al., 2013a; Ye et al., 2014; Ye et al., 2016; Desai et al., 2017; Wu et al., 2017; Ritchie et al., 2020). Studies showed that FGF21 concentrations are increased in liver tissues at an early stage in human subjects and mouse model along with type 2 diabetes or steatohepatitis. However, when HCC develops, FGF21 protein levels are decreased in liver tissues. FGF21 knockout mice fed a high-fat and high-sucrose diet show significantly worse fibrosis, and 78% of mice develop HCC. By contrast, only 6% of WT mice develop HCC. The loss of FGF21 protein in the liver is associated with hyperproliferation and aberrant p53 and TGF-β/Smad signaling during the development of HCC (Liu et al., 2016b). Other studies showed that forced expression of FGF21 could delay the initiation of chemically induced hepatocarcinogenesis, implying the potential anticancer properties of FGF21 (Huang et al., 2006). Related studies indicated that FGF21 is required to limit the progression of HCC carcinogenetic transformation during metabolic liver injury in diabetic subjects, which mainly function at the stage of HCC initiation (Zhang et al., 2015; Singhal et al., 2018). Moreover, Wu L et al. found similar results in patients with CHB. Their results showed that serum FGF21 in CHB patients exhibited a dramatic increase with the occurrence of ACLF and in CHB patients who developed HCC (Wu et al., 2017). Additionally, the high expression of FGF21, FGF19, and FGFR4 is significantly associated with better survival in a multivariate analysis with potential prognostic factors (Yoo et al., 2017). The above studies indicated that FGF21 may be a useful biomarker in monitoring tumorigenesis and evaluating the survival of patients with liver-related diseases/HCC.

### Other FGFS

Besides the above FGFs, other FGFs are also involved in the development and progression of HCC. Overexpressed FGFs, including FGF4, FGF5, FGF9, and FGF22, were detected in HCC tumors but not in samples of cirrhotic tissues (Mas et al., 2007). FGF5 and FGF9 activate FGFR1c with HS in human HCC. FGF5 functions as a major target of miR-188–5p, and its restoration could reverse the inhibitory action of miR-188–5p on HCC cell proliferation and metastasis (Fang et al., 2015). Similarly, FGF9 is as a target of miR-140–5p, and its overexpression attenuates the effect of miR-140–5p on HCC growth and metastasis (Yang et al., 2013b).

Additionally, indirect evidence showed that FGF5 knockout mice could render NASH, which will provide tumor microenvironment and further induce HCC (Hanaka et al., 2014). Missiaglia et al.’s study show that the FGF13 gene was significantly associated with the occurrence of liver metastasis and shorter disease-free survival (Missiaglia et al., 2010). Further study is needed to confirm the underlying relationship.

### FGFRS

The FGFR family consists of FGFR1–4, which are highly conserved transmembrane tyrosine kinases receptors. FGFs transduce a series of biology activity by binding with FGFRs, HSPGs, and klotho type co-receptors. Evidence shows that FGF/FGFR signaling is involved in HCC development and progression, even in cancer treatment. Preclinical data have demonstrated that nearly 50% of HCC were directly or indirectly caused by FGF/FGFR abnormality. The signaling axis of FGF/FGFR is a tissue-specific manner based on the interaction of FGFs, FGFRs, HSPGs, and klotho type co-receptors. Consistent with the high expression levels of FGFR3 and FGFR4 in the liver, the overexpression of FGFR3 and/or FGFR4 was detected in the majority of HCC cases compared with the relatively rare upregulation of FGFR1 and/or FGFR2 (Cappellen et al., 1999; Paur et al., 2015).

FGFR4 is a human hallmark in the study of HCC disease mechanism and drug development owing to its innate advantages. The liver uniquely possesses a complete FGFR4 activating machinery, including FGFR4, FGF19, and KLB, and the specific structure of FGFR4 could be distinguished from that of other FGFRs. FGFR4 overexpression has been found in 30% to almost 50% of HCC tissues (Desnoyers et al., 2008; French et al., 2012; Raja et al., 2019). Two different FGFR4 gene polymorphisms have been associated with increased levels of *a*-fetoprotein in HCC patients (Ho et al., 2009; Yang et al., 2012b; Sheu et al., 2015; Xie et al., 2015). FGFR4 modulates downstream pathways, such as PI3K/AKT and RAS/RAF/MAPK, which are predominantly involved in tumor proliferation and anti-apoptosis. As discussed in the FGF19 section, the abnormality of FGF19-FGFR4-KLB is involved in HCC cell proliferation, survival, EMT, migration, and invasion (Ho et al., 2009; Yang et al., 2012b; French et al., 2012; Lin and Desnoyers, 2012; Liu et al., 2015b; Gu et al., 2015; Sheu et al., 2015; Lin et al., 2019b). FGF19 or FGFR4 functions as a potential therapeutic target for the treatment of HCC patients, which is an active topic in the field of clinical liver oncology (Zhong et al., 2014; Hagel et al., 2015; Repana and Ross, 2015; Sheu et al., 2015; Gao et al., 2017; Joshi et al., 2017; Cui et al., 2018; Kim et al., 2019a; Hatlen et al., 2019; Subbiah and Pal, 2019; Weiss et al., 2019).

The roles of other FGFRs on HCC have been explored. FGFR1 promotes HCC progression and is targeted by a series of microRNAs. Studies showed that the polymorphisms of FGFR1 are related to HBV-related HCC, but they do not have an independent role in tumorigenesis and progression (Wang et al., 2013; Xie et al., 2015). The high expression of FGFR2 induced by FGF7 stimulation is correlated with poor pathologic differentiation, which might increase the incidence of HCC recurrence. FGFR2 fusion mutations are reported in 13–20% of patients with intrahepatic cholangiocarcinoma (Harimoto et al., 2010; Chen et al., 2013; Jun et al., 2020). Interestingly, FGFR2-IIIb expression in HCC tissues and cell lines was lower than that in primary human hepatocytes and nontumorous tissue, and reduced expression of FGFR2IIIb induces a more aggressive growth of HCC (Amann et al., 2010). The role of FGFR2 in HCC is controversial and needs to be further determined. In cancers, FGFR3-mediating signals are often activated by manifold mechanisms, such as activating receptor mutations, translocations, altered splicing, upregulation of FGFs and/or FGFR3, and defects in negative feedback loops. The various mechanisms were reported to be associated with the development and progression of different kinds of cancers, including HCC. Bettina Grasl-Kraupp et al. found that the level of at least one of the two FGFR3 subtypes on the surface of tumor cells is significantly increased in 50% of HCC cases. The concentration of FGFR3 in tumor tissue is positively correlated with the primary tumor size and the recurrence probability. Other previous works identified that FGFR3 overexpression is correlated with lung metastasis and angiogenesis of HCC (Paur et al., 2015; Li et al., 2016a; Liu et al., 2016a; Zhuang et al., 2018).

### Hepatocellular Carcinoma Therapeutics Targeted to FGFRS

Considering the established roles of aberrant FGF/FGFR signaling in liver cancer oncogenesis, inhibitors of the FGF/FGFR signaling axis may be promising for HCC treatment, which slow or halt HCC tumor growth, target angiogenesis and metastasis, and reverse acquired resistance to anticancer agents. The development of FGFR inhibitors started from the earliest multi-target inhibitors to pan-FGFR inhibitors and then to selective FGFR4 inhibitors and irreversible FGFR4 inhibitors (French et al., 2012; Shen et al., 2013; Katoh and Nakagama, 2014; Mellor, 2014; Choi et al., 2015; Joshi et al., 2017; Ettrich and Seufferlein, 2018; Spallanzani et al., 2018; Kim et al., 2019a; Lin et al., 2019a; Doycheva and Thuluvath, 2019; Hatlen et al., 2019; Weiss et al., 2019).

Sorafenib is a landmark in the field of targeted therapy for liver cancer, which is the first approved targeted therapy for HCC and was approved by the FDA in 2007. It is an oral multi-target tyrosine kinase inhibitor with targets including CRAF, BRAF and vascular endothelial growth factor receptor (VEGFR1/2/3) and platelet-derived growth factor receptor (PDGFR) and other tyrosine kinase receptors (KIT, FLT-3, RET, RET). Moreover, for intrahepatic cholangiocarcinoma (ICC), which belong to a type of primary carcinoma of the liver, with FGFR2 gene fusion, sorafenib has a preferable clinical treatment effect (Ying et al., 2019). Sorafenib is currently suitable for the first-line treatment of inoperable or metastatic advanced HCC. Pemigatinib is the first targeted therapeutic drug for intrahepatic cholangiocarcinoma, and was approved by FDA in april 2020. According to the latest 2020 National Comprehensive *Cancer* Network (NCCN) guidelines (United States), if FGFR2 gene fusion or rearrangement is clinically detected in cholangiocarcinoma, the targeted drug pemigatinib can be used for treatment, and there is a favorable response (Abou-Alfa et al., 2020; Romero, 2020).

In addition, according to the 2020 Consensus for clinical application of molecular diagnosis on hepatobiliary carcinoma (China), FGF19 gene in HCC often exhibits copy number amplification, which is closely related to the occurrence and development of HCC (Lu, 2020). The highly selective FGFR4 inhibitors, such as H3B-6527, can significantly inhibit HCC cell proliferation and benefit patients with mutations in the FGF19 signaling pathway. Moreover, FGF/FGFR gene mutation, amplification or gene fusion will cause the continuous activation of FGFR and promote the progression of many tumors. FGFR1-3 gene mutations can be detected in 11% of intrahepatic cholangiocarcinoma (ICC) (Javle et al., 2016), FGFR2 gene fusion was detected in 11–45% of cholangiocarcinoma (CCA), and the common fusion forms mainly include FGFR2-ZMYM4, FGFR2-BICC1 fusion, *etc* (Saha et al., 2016). Furthermore, it should also pay attention to hyperprogressive disease (HPD) in tumor immunotherapy of HCC. The data shows that the incidence of HPD in tumor immunotherapy is about 10% (Champiat et al., 2017; Ferrara et al., 2018), and the gene amplification of MDM2, MDM4, EGFR and 11q13 (including CCND1, FGF3, FGF4, FGF19) may be related to tumor immunotherapy HPD (Kato et al., 2017), the molecular mechanisms of HPD and the relative predictive biomarkers, etc., need further research.

As shown in Table 1, there are several clinical trials of FGF/FGFR inhibitors are in progress, such as multikinase inhibitors anlotinib, regorafenib and nintedanib, pan-FGFR inhibitors erdafitinib, futibatinib, AZD4547, LY2874455. More importantly, FGF19 signaling through the FGFR4/β-klotho receptor complex has been shown to be a key driver of growth and survival in hepatocellular carcinoma, which makes selective FGFR4 inhibition an attractive therapeutic opportunity. FGFR4 specific targeted drugs, including reversible and irreversible inhibitors, are being developed and researched. FGFR4 selective reversible inhibitors, such as H3B-6527, roblitinib (FGF401), ABSK-011 and ICP-105, bind to the FGFR4 kinase domain in a reversible covalent manner and inhibit the progression of HCC; Whereas, fisogatinib (BLU-554) and BLU-9931 bind to FGFR4 in an irreversible manner. However, most of these agents are still in early phase of clinical trials, and still have a long way to go before they can be widely used in clinical. The success of these therapies requires a comprehensive research and specific selection of patients whose tumors appear aberrant of the FGF/FGFR pathway.

**TABLE 1:** Classification and representatives of FGFR inhibitors for HCC.

Classification	Drug name (alternative name)	Organization	Drug target	Phase	Clinical trial Id
Multikinase inhibitors	Anlotinib	Chia tai-tianqing	VEGFR1/2/3, FGFR, PDGFR, KIT	Phase 3	NCT04344158
	Regorafenib	Bayer	VEGFR, FGFR, tie-1/2, PDGFR, KIT, RAF-1, BRAF, ^600^V, RET	Phase 2	NCT04696055
	Nintedanib (BIBF1120)	Boehringer-ingelheim	FGFR, VEGFR, PDGFR	Phase 1	NCT01594125
				Phase 2	NCT00987935
				Phase 1/2	NCT01004003
Pan-FGFR inhibitors	Erdafitinib (JNJ42756493)	Janssen	FGFR1-4	Phase1/2	NCT02421185
				Phase 1/2	NCT02052778
	Futibatinib (TAS-120)	Taiho	FGFR1-4	Phase 3	NCT04093362
	AZD4547	AstraZeneca	FGFR1-4	Phase 2	NCT02465060
	LY2874455	Eli lilly	FGFR1-4	Phase 1	NCT01212107
FGFR4 selective reversible inhibitors	H3B-6527	H3 biomedicine	FGFR4-specific	Phase 1	NCT02834780
	Roblitinib (FGF401)	Novartis	FGFR4-specific	Phase 1/2	NCT02325739
	ABSK-011	Abbisko	FGFR4-specific	Preclinical	—
	ICP-105	InnoCare	FGFR4-specific	Phase 1	NCT03642834
FGFR4 selective irreversible inhibitors	Fisogatinib (BLU-554)	CStone, blueprint	FGFR4, FGF19	Phase 1/2	NCT04194801
				Phase 1	NCT02508467
	BLU-9931	Blueprint	FGFR4-specific	Preclinical	—

## Conclusion

The FGF/FGFR axis plays a vital role in the development and treatment of HCC. Particularly, the FGF19-FGFR4-KLB signaling system has been recognized as the main driver of hepatocarcinogenesis, and several FGFR4-specific inhibitors are being tested in clinical trials. These findings and clinical trials will be utilized to unveil the importance of the FGF/FGFR family on the HCC mechanism and speed up the development of “precise medicine” strategies for HCC treatment.
